# Pulmonary vascular adaptations to hypoxia in elite breath-hold divers

**DOI:** 10.3389/fphys.2024.1296537

**Published:** 2024-07-31

**Authors:** Thomas Kjeld, Anders Brenøe Isbrand, Henrik Christian Arendrup, Jens Højberg, Jacob Bejder, Thomas O. Krag, John Vissing, Lars Poulsen Tolbod, Johannes Hendrik Harms, Lars Christian Gormsen, Dan Fuglø, Egon Godthaab Hansen

**Affiliations:** ^1^ Department of Anesthesiology, Herlev Hospital, University of Copenhagen, Copenhagen, Denmark; ^2^ Department of Clinical Medicine, Faculty of Medicine, University of Copenhagen, Copenhagen, Denmark; ^3^ Department of Cardiothoracic Anesthesiology, Rigshospitalet, University of Copenhagen, Copenhagen, Denmark; ^4^ Department of Nutrition, Exercise and Sport (NEXS), University of Copenhagen, Copenhagen, Denmark; ^5^ Department of Neurology, Rigshospitalet, University of Copenhagen, Copenhagen, Denmark; ^6^ Department of Nuclear Medicine & PET Centre, Aarhus University Hospital, Aarhus, Denmark; ^7^ Department of Nuclear Medicine, Herlev Hospital, University of Copenhagen, Copenhagen, Denmark

**Keywords:** cardiac MR (CMR), cardiac PET/CT, echocardiagraphy, freediving, cardiac output

## Abstract

**Introduction:**

Elite breath-hold divers (BHD) possess several oxygen conserving adaptations to endure long dives similar to diving mammals. During dives, Bottlenose Dolphins may increase the alveolar ventilation (V_A_) to perfusion (Q) ratio to increase alveolar oxygen delivery. We hypothesized that BHD possess similar adaptive mechanisms during apnea.

**Methods and results:**

Pulmonary blood volume (PBV) was determined by echocardiography, ^15^O-H_2_O PET/CT, and cardiac MRi, (n = 6) during and after maximum apneas. Pulmonary function was determined by body box spirometry and compared to matched controls. After 2 min of apnea, the PBV determined by echocardiography and ^15^O-H_2_O-PET/CT decreased by 26% and 41%, respectively. After 4 min of apnea, the PBV assessed by echocardiography and cardiac MRi decreased by 48% and 67%, respectively (n = 6). Fractional saturation (F)O_2_Hb determined by arterial blood-gas-analyses collected after warm-up and a 5-minute pool-apnea (n = 9) decreased by 43%. Compared to matched controls (n = 8), spirometry revealed a higher total and alveolar-lung-capacity in BHD (n = 9), but a lower diffusion-constant.

**Conclusion:**

Our results contrast with previous studies, that demonstrated similar lung gas transfer in BHD and matched controls. We conclude that elite BHD 1) have a lower diffusion constant than matched controls, and 2) gradually decrease PBV during apnea and in turn increase V_A_/Q to increase alveolar oxygen delivery during maximum apnea. We suggest that BHD possess pulmonary adaptations similar to diving mammals to tolerate decreasing tissue oxygenation.

**New and noteworthy:**

This manuscript addresses novel knowledge on tolerance to hypoxia during diving, which is shared by elite breath-hold divers and adult diving mammals: Our study indicates that elite breath-hold divers gradually decrease pulmonary blood volume and in turn increase VA/Q, to increase alveolar oxygen delivery during maximum apnea to tolerate decreasing oxygen levels similar to the Bottlenose Dolphin.

## Introduction

Diving mammals like dolphins with comparable diving duration as human elite breath-hold divers (BHD) (http://www.freedive-earth.com/aida-freediving-world-records), also have lung volumes comparable to terrestrial mammals (Snyder, 1983). Dolphins dive following inspiration and use the lungs as an oxygen store (Snyder, 1983), similar to breath hold divers (BHD), who also increase lung oxygen stores by glossopharyngeal insufflation (GPI), and hereby increases the ventilation to perfusion ratio (V_A_/Q ratio) (Eichinger et al., 2008). However, oxygen stores are limited and to compensate for this during longer dives, the Bottlenose Dolphin may also be able to increase the V_A_/Q ratio by decreasing lung perfusion, hence favoring the exchange of alveolar oxygen over pulmonary capillary carbon dioxide (Fahlman, Jensen, Tyack, & Wells, 2018; Garcia, Moore, & Fahlman, 2018). Previous studies of pulmonary blood volume after 4 min of apnea in BHD concluded that GPI causes lung vessel compression resulting in a decrease in pulmonary blood volume (Mijacika et al., 2017a; Mijacika et al., 2017b). However, the proposed adaptation of the Bottlenose Dolphin described above indicates, that pulmonary blood volume may be redistributed to increase V_A_/Q, and hereby increasing alveolar gas exchange to compensate for decreasing oxygen stores during dives. The question is whether this also could be the case in BHD, who possess several adaptations to hypoxia, similar to adult diving mammals (Kjeld et al., 2018; Kjeld et al., 2021c; Chicco et al., 2014).

We hypothesized that human BHD with self-reported apneas of more than 5 minutes would have similar adaptations towards hypoxia as diving mammals by gradually decreasing pulmonary blood volume as part of the diving response. Hence, this study aimed to demonstrate these hypothesized adaptations in BHD, and as pulmonary blood volume can be determined by multiplying pulmonary transit time and cardiac output, these parameters were quantified using the imaging modalities echocardiography, ^15^O-H_2_O-PET/CT and cardiac MRi (Harms et al., 2015; Jorstig, Waldenborg, Liden, & Thunberg, 2017; Kaul, Tei, Hopkins, & Shah, 1984; Margossian et al., 2009) during maximum dry apneas, whereas the oxygen binding properties of the hemoglobin was quantified using arterial blood gas analyses before, during and after maximum pool apneas.

## Methods

This study included seventeen healthy/non-medicated male non-smoking subjects and was approved by the Regional Ethics Committee of Copenhagen (H-1-2013-060). All investigations were conducted according to the principles expressed in the Declaration of Helsinki. Informed consent, written and oral, were obtained from the participants. Nine subjects were divers (age 42 ± 3 years) and eight were judo athletes matched for morphometric variables (age, height, weight, body mass index) and maximal oxygen uptake (VO_2_max, Supplementary Table 1) were chosen for comparison. Judo athletes were chosen as controls, as they do aerobic exercise in contrast to BHD.

All BHD included in this study had a recent competition verified maximum apnea of at least 5 minutes. The BHD all ranked among national top 10, three of the participating BHD ranked among World top 10, and one was a 2016 outdoor free-diving World champion, while one reached third place at the same Championship (no limit depth competition), and one was a World record holder.

All the matched controls were either judo or jiu-jitsu black belts, all were medalists at national championships, and all except one were active fighters.

### VO_2_max and body box spirometry

Subjects completed a physical fitness test to determine maximal oxygen uptake *V*O_2_max (Supplementary Table 1): participants completed a standardized warm-up followed by an incremental cycle test (Monark, Varberg, Sweden) starting at a workload of 150 W and increasing by 25 W every minute until exhaustion (Astrand and Ryhming, 1954). The highest recorded (Quark, Cosmed, Rome, Italy) 30 s average oxygen uptake (VO_2_) during the test was defined as VO_2_max. For the recognition of true VO_2_max, three of five criteria had to be met: individual perception of exhaustion, respiratory exchange ratio >1.15, plateau of the VO_2_ curve, heart rate approaching the age-predicted maximum and inability to maintain a pedaling frequency above 70 rpm To determine pulmonary function, subjects completed Body box spirometry according to the guidelines of the European Respiratory Society (Stanojevic et al., 2022; Stanojevic et al., 2022) (Master Screen Body, Jaeger, Würzburg, Germany, Table 1).

**TABLE 1.** Pulmonary function measurements (Body box spirometry).

	Breath-hold divers	Controls	p
Number of subjects	9 males	8 males	N/A
Forced Vital Capacity, FVC (L)	7.0 ± 0.7	6.0 ± 0.6^#^	0.003
FVC _% predicted_	134.1 ± 6.8	112.5 ± 9.0^#^	<0.001
Forced Expiratory Volume, FEV1 (L)	5.3 ± 0.6	4.6 ± 0.8	NS
FEV1_% predicted_	123.7 ± 8.7	105.7 ± 14.1^*^	0.009
FEV1/FVC (%)	75.3 ± 5.8	77.2 ± 2.7	NS
FEV1/FVC _% predicted_	94.3 ± 6.7	95.4 ± 2.9	NS
Intrathoracic gas volume (L)	3.9 ± 1.0	3.9 ± 0.2	NS
Intrathoracic gas volume _% predicted_	109 ± 0.8	111 ± 4	NS
Expiratory reserve volume (L)	2.0 ± 0.7	1.9 ± 0.2	NS
Expiratory reserve volume _% predicted_	131 ± 0.4	118 ± 10	NS
Vital capacity (L)	6.8 ± 0.2	5.8 ± 0.1^*^	0.006
Vital capacity _% predicted_	123 ± 3	105 ± 3^#^	<0.001
Total Lung Capacity (L)	8.7 ± 0.3	7.8 ± 0.1^*^	0.012
Total Lung Capacity _% predicted_	116 ± 3	103 ± 2	0.051
Residual volume (L)	2.2 ± 0.2	2.0 ± 0.1	NS
Residual volume _% predicted_	103 ± 6	104 ± 5	NS
Diffusion capacity for CO D_LCO_ (mmol/(min*kPa))	11.8 ± 0.7	12.3 ± 0.6	NS
D_LCO_ _% predicted_	100 ± 4	102 ± 4	NS
Diffusion constant, K_CO_ (mmol/(min*kPa*L))	1.4 ± 0.07	1.7 ± 0.06^*^	0.012
K_CO % predicted_	89 ± 4	106 ± 3^*^	0.021
Alveolar volume (L)	8.7 ± 0.3	7.3 ± 0.2^#^	<0.001
Alveolar Volume _% predicted_	115 ± 2	98 ± 2^#^	<0.001

CO: Carbon monoxide. Values are mean ± Standard error of mean. #p < 0.005. *p < 0.05. NS: Not statistically significant.

### Pool apneas

The nine elite BHD were instructed to refrain from intake of caffeine and to be fasting for at least 6 h before the pool apneas as described previously (Kjeld et al., 2021c). Any strenuous physical activity was discouraged for at least 1 day before the experiment.

A 1.1 mm, 20-gauge catheter was inserted in the radial artery of the non-dominant arm with connection to continuous flow of saline (3 ml/h; Baxter, Uden, the Netherlands) for collection of blood gases. Blood gas analyses were performed immediately after sampling, using an automated self-calibrating blood gas machine (ABL 835, Radiometer, Copenhagen, Denmark as described previously (Kjeld et al., 2021c) analyzing arterial hemoglobin, hematocrit, met-hemoglobin, and the fraction of oxygenated hemoglobin (FO_2_Hb) with a high degree of accuracy (Weber et al., 2000).

The BHD performed head-immersed maximal static apnea after glossopharyngeal insufflation (GPI) (Seccombe et al., 2006) in a 28° Celsius, 0.8 m deep indoor pool after a warm-up of three consecutive apneas to maximize the diving response (Kjeld, Pott, & Secher, 2009; Kjeld et al., 2021a; Kjeld et al., 2021c). The subjects were instructed to give a sign with their index finger just before terminating maximum apnea, so blood gas samples could be collected just before breathing was resumed. Blood gases were also collected after 4 min of apnea.

### ^15^O-H_2_O-PET/CT: imaging protocol and image reconstruction

The six subjects with the longest pool maximum apneas (378 ± 78 s), were recruited for the ^15^O-H_2_O-PET/CT study. The participants in the ^15^O-H_2_O-PET/CT sub-study were required to be able to hold their breath for 5 minutes while lying in the PET/CT scanner in the supine position with arms raised above the head. They were instructed to refrain from intake of caffeine and chocolate, to refrain from strenuous physical exercise for one day and to be fasting for at least 6 h before the study.

**FIGURE 1:**
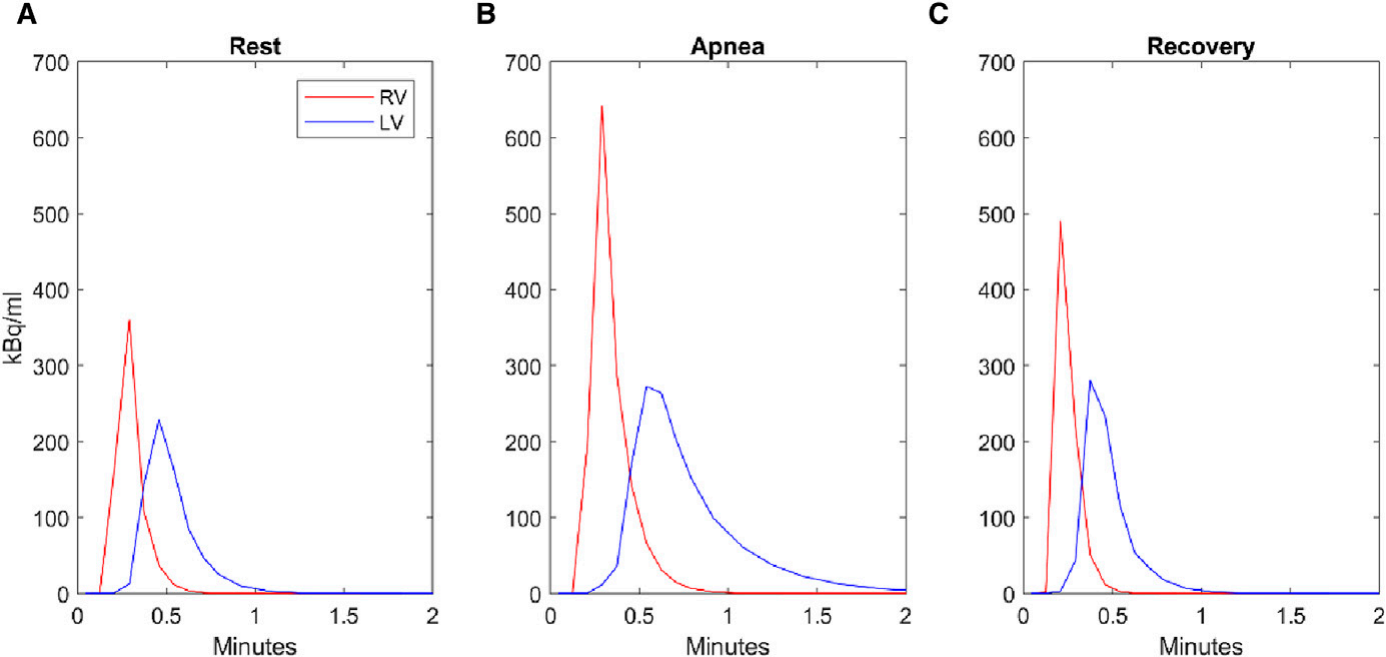
Calculations of ^15^O-H_2_O-PET/CT determined pulmonary blood volume. First-pass time-activity curves extracted from 3 consecutive 4-min ^15^O-H_2_O/PET-CT scans on the same subject (only the 2 first minutes are shown). The mean transit time from the right ventricle to the left ventricle cavity increases from 13s at rest **(A)** to 22s during apnea (initiated 2 min prior to the scan) **(B)** and returns to 13s during recovery **(C)**.

To evaluate whether pulmonary blood volume would decrease during apnea, images were obtained at rest, after 2 min or after 4 min of apnea, and in the period following apnea. To minimize radiation exposure, images were collected in separate groups, as described below:

^15^O-H_2_O-PET/CT data were obtained in list mode on a GE Discovery MI Digital Ready PET/CT system (GE, Milwaukee, WI, USA). The participants underwent three image acquisitions: 1) at rest, 2) during hyperemia induced by a dry static apnea after GPI (Seccombe et al., 2006) and after a warm-up of three consecutive apneas to maximize the diving response (Kjeld et al., 2009; Kjeld et al., 2021a), and 3) third in the recovery phase 4 min after the apnea (Figure 1). For each acquisition, 400 MBq ^15^H_2_O was administered intravenously in an antecubital vein as a single bolus using an automated injection system (Medrad Stellant, Bayer, Leverkusen, Germany). For the apnea acquisition, ^15^O-H_2_O was infused after 2 minutes (n = 3) or 4 minutes (n = 3) after initiation of the apnea with acquisition of data for at least an additional 2 minutes (Figure 1). Average apnea duration was 378 ± 77 s. For each PET scan, an attenuation CT was performed at time points allowing for correct co-registration of the PET and CT images. The resting and recovery attenuation correction CT scans were thus performed immediately prior to and after the PET scans, whereas the apnea attenuation correction CT was performed during warm-up mimicking maximal apnea. The presented ^15^O-H_2_O-PET/CT data are post hoc analysis of data collected during the study by Kjeld T. et al 2021 (Kjeld et al., 2021c).

### ^15^O-H_2_O-PET/CT: Image analysis

Cardiac output and mean transit-times were quantified by ^15^O-H_2_O-PET/CT on a GE Discovery MI Digital Ready PET/CT system (GE, Milwaukee, WI, USA) at time points as specified above and as described previously (Kjeld et al., 2021c). The images were reconstructed in a 3.27 × 3.27 × 3.27 mm matrix utilizing all normal corrections (attenuation, scatter, dead time and random) and the Vue Point FX-S reconstruction algorithm. For subsequent analysis, the dynamic scan was divided into 21 frames (1 × 10, 8 × 5, 4 × 10, 2 × 15, 3 × 20 and 2 × 30 seconds). Curves showing the activity of ^15^O-H_2_O as a function of time for the superior vena cava, right ventricle, left ventricle and ascending aorta were extracted automatically using in-house developed software. Cardiac output and mean transit-times were calculated from the time-activity curves using the indicator dilution principle as previously described (Harms et al., 2015; Harms and Sorensen, 2020). In short, for each region, the first-pass was isolated in the time-activity curves using down-slope fitting. The forward cardiac output was then calculated was the total injected activity of ^15^O-H_2_O divided by area under the first pass curve of the left ventricle, whereas mean transit times between two regions were calculated as the time between centroids of the two regions first pass curves. The blood volume between two regions were calculated as the product of the forward cardiac output and the mean transit time.

### Cardiac magnetic resonance imaging (CMRi): image acquisition

For one day prior to the study, the same six subjects as recruited for the ^15^O-H_2_O-PET/CT sub-study refrained from physical exercise and consumption of caffeine. To minimize contrast exposure, images were only obtained after 4 min of apnea to evaluate pulmonary blood volume. Imaging was performed in a 1.5 T MR imaging system (Achieva, Philips Medical System, The Netherlands) after a warm-up of three consecutive apneas to maximize the diving response (Kjeld et al., 2009). Cine images were acquired only after 4 min of dry static apnea after GPI (Kjeld et al., 2021c), and the pulmonary blood volume estimated by ^15^O-H_2_O-PET/CT at rest were used as baseline for comparison of pulmonary blood volume assessed by CMRi, to spare the subjects to be exposed to contrast two times during CMRi study. Images were collected shortly before end of GPI apnea before breathing, and subjects were instructed to stay as calm as possible during imaging to avoid imaging artefacts. Cardiac chamber volumes and function were analyzed in short axis images acquired using a retrospectively ECG-gated steady-state free precession sequence reconstructed to 25 phases covering the entire cardiac cycle. The following typical settings were used: TR/TE 3.3/1.6 ms, flip angle 60°; and spatial resolution 1.3 × 1.3 × 8 mm^3^. For calculation of pulmonary transit time, a first-pass perfusion imaging sequence was used at the end of the GPI apnea after a bolus of gadolinium contrast was injected through a median cubital vein (Nelsson et al., 2021). Time resolved imaging was performed using a balanced turbo field echo sequence in the short-axis plane with the following typical parameters: TR/TE 2.6/1.3 ms, flip angle 50°, and a spatial resolution of 1.4 × 1.4 mm^2^. The temporal resolution was 1 acquisition per heartbeat and the total scan time for the first-pass perfusion sequence was 1-2 minutes. The presented CMRi data are post hoc analysis of data collected during the study by Kjeld et al. 2021 (Kjeld et al. 2021c).

### Cardiac magnetic resonance imaging (CMRi): Image analysis

**FIGURE 2:**
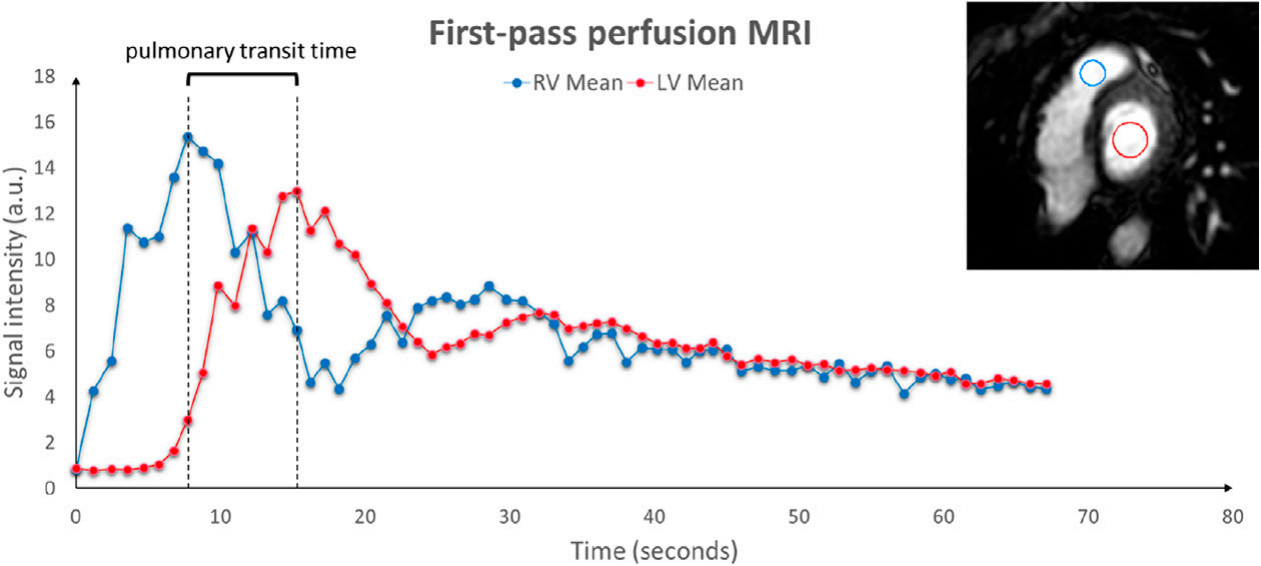
Example of cardiac MRi determined pulmonary transit time. *X*-axis: time in seconds after 4 min of apnea and concomitant contrast infusion. *Y*-axis signal intensity. RV: Right ventricle. LV: left ventricle.

Left and right ventricular volumes were determined in end-diastole and end-systole and used to determine the influence of apnea on cardiac volumes, function, filling, and individual chamber outflow as described previously (Kjeld et al., 2021c). Data were analyzed by two clinical physiologists: one experienced level 3 nuclear physiologist and one level one cardiac magnetic resonance cardiologist using dedicated software (Segment Medviso, Sweden for chamber volumes and Intellispace, Philips, The Netherlands for pulmonary transit time (PTT)) carefully avoiding image artefacts especially during involuntary breathing movements. Stroke volume was computed as the difference between end diastolic volume and end systolic volume. Cardiac outputs were computed as SV multiplied by heart rate. PTT was determined in the most basal first pass perfusion (FPP) short axis slice from the right ventricle to the left ventricle FPP (Nelsson et al., 2021). Regions of interest were placed in the right ventricular outflow tract and the left ventricle, and to compensate for motion they were drawn manually in each time frame of the FPP images. The transit time was calculated using time-intensity curves for the two regions of interest and measuring the time difference between peaks. As peak time was not always well delineated, we also performed the same calculations using the midpoint of the timeintensity curves (Figure 2). Pulmonary blood volumes were computed as right ventricle cardiac output multiplied by PTT (Nelsson et al., 2021).

### Echocardiography: image acquisition

**FIGURE 3:**
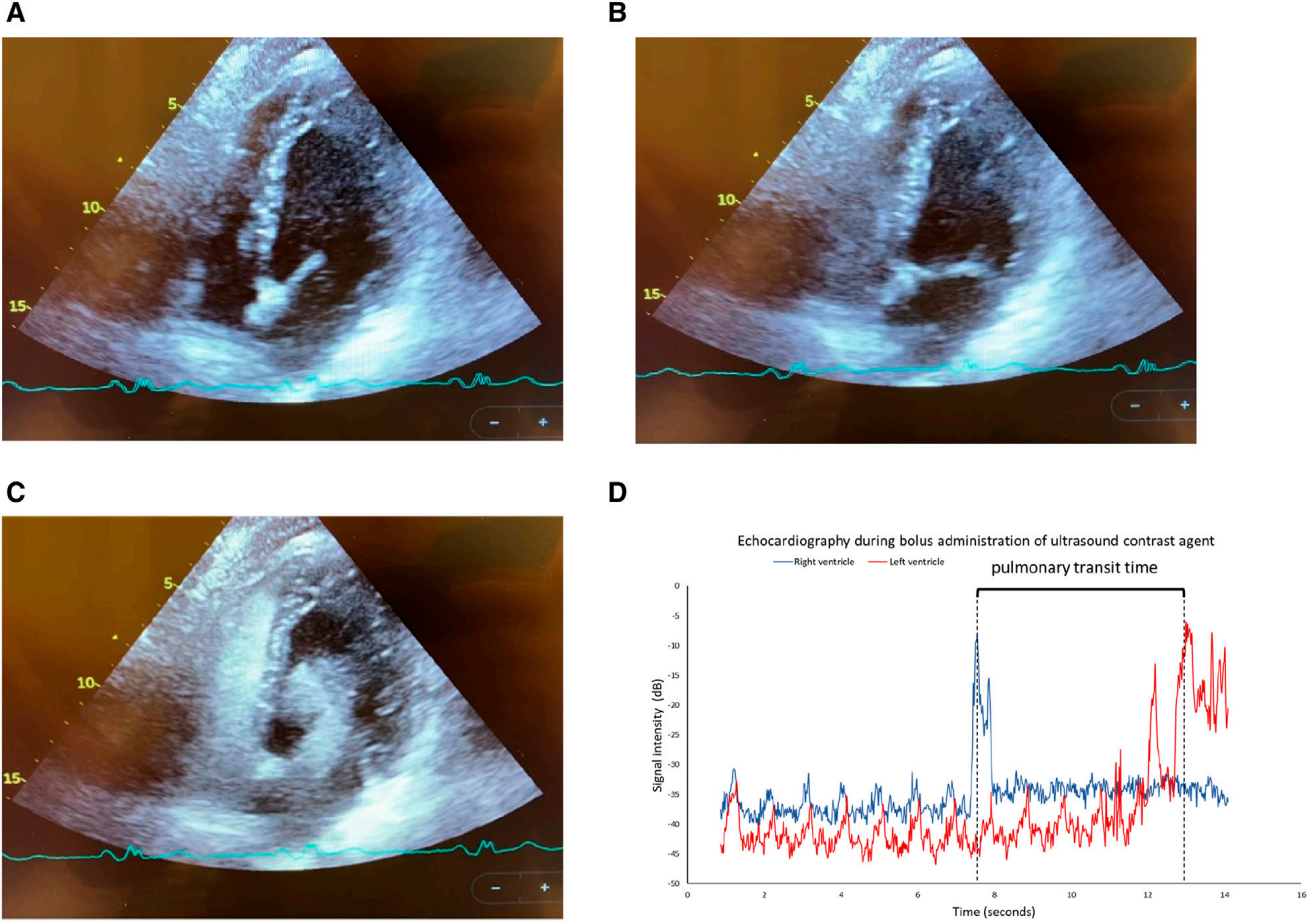
Example of pulmonary transit time determined by echocardiography after 4 min of apnea. RV: Right ventricle. LV: left ventricle. 4D displays ultrasonic determined pulmonary transit time using time-activity curves in RV and LV after a bolus of contrast agent. **(A)** before contrast; **(B)** contrast in the RV; **(C)** contrast in the LV; **(D)** pulmonary transit time using time-activity curves in the RV and LV.

For one day prior to the study, the same six BHD as recruited for the ^15^O-H_2_O-PET/CT and CMRi sub-studies refrained from physical exercise and were fasting for 4 hours. The participants underwent three image acquisitions: 1) at rest, 2) during hyperemia induced by a dry static apnea after GPI (Seccombe et al., 2006) of 2 min duration and after a warm-up of three consecutive apneas to maximize the diving response (Kjeld et al., 2009; Kjeld et al., 2021a), and 3) during hyperemia induced by a dry static apnea after GPI (Seccombe et al., 2006) of 4 min duration and after a warm-up of three consecutive apneas to maximize the diving response (Kjeld et al., 2009; Kjeld et al., 2021a) (Figure 3). For each acquisition, 1.5 ml of Optison^®^ was administered intravenously in an antecubital vein as a single bolus (Supplementary Video S1). Before each image acquisition during apnea, a test scan was performed after GPI to find focus for placing the ultrasound probe for imaging on the subject.

6 control subjects were recruited for similar echocardiographic measurements as the BHD underwent, but only at rest.

### Echocardiography: image analysis

Left and right ventricular volumes were determined in end-diastole and end-systole and used to determine the influence of apnea on cardiac volume, function, filling, and individual chamber outflow as described previously (Kjeld et al., 2015). Data were analyzed by two clinicians, one experienced nuclear physiologist and one level three cardiologist using dedicated software (Echopac GE^®^, USA for chamber volumes). Pulmonary transit time (PTT) was assessed by measuring the time from contrast passing from the right ventricle to the left ventricle in a four-chamber view. Stroke volume was computed as the difference between end diastolic volume and end systolic volume. Cardiac output was computed as SV multiplied by heart rate and pulmonary blood volume was calculated by multiplying left ventricle cardiac output by PTT.

### Statistical analysis

Variables are presented as mean ± standard error of the mean (SEM). Data were analyzed by Sigma-Plot^®^ using one-way repeated measures ANOVA. The Holm-Sidak method *post hoc* was used to evaluate differences between the collected data during rest, apnea, and recovery. A p-value <0.05 was considered statistically significant.

## Results

Compared to controls, BHD had higher predicted FEV1, vital capacity and alveolar volume (Table 1). Total lung capacity tended to be higher in BHD as compared to controls only borderline significant (p = 0.051). The diffusion capacity for carbon monoxide was not different between BHD and controls, but BHD had lower diffusion constant than the controls (diffusion capacity per alveolar volume).

During apnea overall ^15^O-H_2_O-PET/CT assessed PTT from right ventricle to left ventricle increased from 11.0 ± 0.8 s to 16.5 ± 1.8 s (p = 0.002), Expressing PTT in the number of heart beats needed for the tracer to pass from right ventricle to left ventricle, this corresponds to an increase from 10 ± 1 beats to 14 ± 1 beats (p = 0.005). PTT from vena cava to ascending aorta increased from 12.0 ± 1 s to 17.2 ± 2.0 s (p = 0.003), corresponding to an increase from 11 ± 1 beats to 15 ± 1 beats (*p* = 0.009). Sub analyses after 2 min of apnea revealed decreased pulmonary blood volume from superior vena cava to ascending aorta: 1295 ± 88 ml to 754 ± 52 ml (n = 3, p = 0.037, Table 2A & 2B). PET-CT data after 4 min of apnea were inconclusive.

**TABLE 2A.** PET-CT evaluated Pulmonary transit time (PTT), pulmonary blood volume vena cava to aorta ascendens (PBV VC to AA), heart rate (HR), cardiac output (CO) at rest and after 2 min of apnea (n = 3 breath hold diver).

	Rest	2 min apnea
RV to LV (s)	11.0 ± 0.8	17.4 ± 2.7^#^
HR (beats/min)	56 ± 4	50 ± 7
LVCO (ml/s)	108.3 ± 13.3	40.0 ± 8.3^*^
PBV (ml)	1295 ± 88	754 ± 52^#^

Values are mean ± SD. *: p ≤ 0.005 compared to rest. #p ≤ 0.05 compared to rest.

**TABLE 2B.** CMR-evaluated Pulmonary transit time (PTT), pulmonary blood volume from the vena cava to the aorta ascendens (PBV VC to AA), heart rate (HR), cardiac output (CO) after 4 min of apnea (n = 5 breath-hold divers.

4 min apnea (CMR)			
PTT RV to LV (s)	7.6 ± 0.8*		
HR (beats/min)	61 ± 7		
RVCO (ml/s)	55 ± 4*	LVCO (ml/s)	81 ± 18
PBV (RV) (ml)	418 ± 6*	PBV (LV) (ml)	589 ± 97

Values are mean ± SD. *: p ≤ 0.005 compared to rest.

The CMR assessed right ventricle cardiac output decreased from 142 ± 9 ml/s at baseline to 55 ± 4 ml/s at the end of apnea, where the CMR measured PTT was 7.6 ± 0.8 s, and hence pulmonary blood volume after 4 min of apnea calculates to 418 ± 60 ml (n = 5, Figure 2). Number of subjects analyzed were 5 BHD, as results in one subject were inconclusive.

BHD and controls had comparable cardiac variables and pulmonary blood volumes assessed by echocardiography at rest (Supplementary material Table 2 & Table 2C). The left ventricle cardiac output assessed by echocardiography decreased from 70.9 ± 8.8 ml/s at baseline to 42.5 ± 10.8 ml/s at the end of 2 min of apnea, while the PTT remained unchanged (5.3 ± 2.3 s at rest and 5.8 ± 2.1 s after 2 min of apnea). Hence, pulmonary blood volume at rest calculates to be 359.2 ± 93.5 and decreased to 265.9 ± 83.6 ml after 2 min of apnea (p < 0.005; Table 2C). After 4 min of apnea, left ventricle cardiac output assessed by echocardiography was 26.5 ± 5.2 ml/s, and the PTT remained unchanged (5.4 ± 1.5 s). Hence, pulmonary blood volume after 4 min of apnea calculates to 171.0 ± 67.2 ml (p < 0.01 compared to rest and after 2 min of apnea; Table 2C; Figure 3).

**TABLE 2C.** Echocardiographically-determined pulmonary transit time (PTT), heart rate (HR), right ventricle cardiac output (LVCO) and pulmonary blood volume (PBV) assessed from the tricuspid valve passage to passage through the mitral valve at rest, after 2 min and 4 min of apnea (n = 6 breath hold divers and 6 controls).

	Controls: Rest	BHD: Rest	BHD: 2 min apnea	BHD: 4 min apnea
PTT (s)	5.0 ± 1.1	5.3 ± 2.3	5.8 ± 2.1	5.4 ± 1.5
HR (beats/min)	57 ± 7	52 ± 5	55 ± 12	55 ± 21
LVCO (ml/s)	62.5 ± 12.4	70.9 ± 8.8	42.5 ± 10.8^*^	26.5 ± 5.2^#^
PBV (ml)	327.9 ± 86.4	359.2 ± 93.5	265.9 ± 83.6^*^	171.0 ± 67.2^#^

Values are mean ± SD. *: *p* ≤ 0.005 compared to rest. #*p* ≤ 0.05 compared to rest and 2 min of apnea.

During the pool apnea of maximum duration (385 ± 70 s) FO_2_Hb decreased from 95.2 ± 0.5% to 53.9 ± 5.9% (p < 0.001; range 79.2 to 36.2%; Table 3).

**TABLE 3.** Arterial blood gas variables of 9 elite BHD at rest, after 4 min apnea and just before the end of maximal apnea (385 ± 70 s).

Variable	Rest	4 min apnea	End apnea
FO_2_Hb/%	95.2 ± 0.5	75.5 ± 3.3 *	53.9 ± 5.9 *
Hemoglobin/mmol/L	9.3 ± 0.1	9.2 ± 0.1	9.4 ± 0.1
Hematocrit	46.0 ± 0.6	45.3 ± 0.6	46.7 ± 0.6
Met-Hemoglobin	0.76 ± 0.03	0.77 ± 0.05	0.74 ± 0.04
pH	7.417 ± 0.029	7.377 ± 0.0364 #	7.38 ± 0.052 #
pCO_2_/kPa	5.19 ± 0.49	6.14 ± 0.78 *	6.70 ± 0.85 *
pO_2_/kPa	12.15 ± 1.45	6.02 ± 1.16 *	4.69 ± 1.63 *
Lactate/mmol/L	1.8 ± 0.5	1.4 ± 0.6 #	1.6 ± 0.9 ¤
Bicarbonate/mmol/L	25.0 ± 1.5	24.7 ± 1.6	24.7 ± 1.4
Base Excess/mmol/L	0.6 ± 1.8	1.4 ± 2.0	1.8 ± 2.5

Values are mean ± Standard Error of the Mean. FO_2_Hb: fraction of oxygenated hemoglobin. *: p < 0.001 compared to baseline. #p < 0.05 compared to baseline. ¤ p = 0.05 compared to baseline.

Hemoglobin, hematocrit, and met-hemoglobin were unchanged during maximum pool apnea (Table 3).

pH, pO_2_ and lactate decreased, whereas pCO_2_, base excess and bicarbonate increased (p < 0.05; Table 3).

The levels of Ferritin, Iron and hemoglobin were not different in BHD as compared to controls (Supplementary Table 1).

## Discussion

The main and novel findings of our study are 1) compared to controls, BHD have higher vital capacity and higher alveolar volume, but a lower diffusion capacity per alveolar volume and diffusion constant than controls. 2) Pulmonary blood volume determined by echocardiography, ^15^O-H_2_O-PET/CT and cardiac MRi decreased gradually after 2 min and 4 min of apnea, respectively, due to a decrease in cardiac output. 3) At end of maximum pool apnea FO_2_Hb was reduced by almost 50%. Hence, our results suggest that BHD may be able to increase V_a_/Q and thereby increase alveolar oxygen delivery. To our knowledge these adaptations to hypoxia have never been demonstrated in humans before.

### Hypoxic tolerance of elite BHD

Our results also confirmed similar findings regarding pH, pCO_2_, pO_2_, base excess, bicarbonate and lactate as we have described previously (Kjeld et al., 2021c), underlining that the subjects in this study are well adapted to apnea diving and tolerance for hypoxia: the subjects tolerated desaturation ∼ to 4.3 kPa similar to diving mammals (Qvist et al., 1986), and as discussed below, the hypoxia during maximum apnea in elite BHD is well-known to be causing decreasing cardiac output (∼ 50% of resting values) (Kjeld et al., 2009; Kjeld et al., 2021a; Kjeld et al., 2021c). Myocardial contractility has been demonstrated to decrease during in vitro hypoxia, which in turn explains the gradually decreasing cardiac output in elite BHD during the hypoxia induced by apnea (Bing, Apstein, & Brooks, 1975). Elite BHD tolerates the above mentioned extreme hypoxia without cardiac ischemia due to similar adaptations as diving mammals (Kjeld et al., 2021c; Kjeld et al., 2015) including lactate metabolization and hence stable glucose levels (Kjeld et al., 2021a), which in turn support cardiac recovery and inotropic activity upon reversing the hypoxia during breathing (Bing et al., 1975; Kjeld et al., 2021c; Kjeld et al., 2021a; Kjeld et al., 2021b; Kjeld et al., 2015). The diving response is maximal after 3-4 min of apnea, in terms of maximum bradycardia and increases in blood pressure (Kjeld et al., 2021a). However, during apnea, the hypoxia progresses continuously (Kjeld et al., 2021a), and the gradually decreasing cardiac output during apnea is therefore likely associated with reduced myocardial contractility due to the progressively developing hypoxia.

### Lung capacity of elite BHD

Free divers have developed techniques to improve their performance. Prior to a dive, they hyperventilate, and from approximately year 2000 competing BHD began systematically to hyperinflate their lungs using GPI, hereby increasing the air available for pressure equilibration and oxygen storage in the tissue by up to 30% (Eichinger et al., 2008; Seccombe et al., 2006). However, the performances of the BHD almost doubled in length, duration, and depth over a decade (http//www.apnearanking.se; Supplementary Table 1). Hence, the extra air volume made available from hyperinflation of the lungs, cannot explain the increase in performances alone.

Tetzlaff et al. demonstrated that BHD had higher than predicted ventilatory flows and volumes and did not differ from control groups with regard to gas transfer, inspiratory muscle strength, and lung compliance. (Tetzlaff et al., 2008). Our study confirms that the overall lung diffusion capacity of BHD did not differ from controls. However, as a difference to the latter study, we found that after adjustment for the higher total lung (and alveolar) volume in BHD, there was an even lower diffusion capacity than in the controls. Notably, it is subsequently not possible to modify or improve the alveolar membrane with regard to permeability by training or hyperinflation, but only to increase the total volume capacity and respiratory function. However, it may be possible to change pulmonary vessel compliance and as such alter the VA/Q relationship by decreasing lung perfusion as the Bottlenose Dolphin may do during dives, to compensate for the decreasing stores of lung oxygen, and this may - at least partly - explain the improved performances of elite BHD (Fahlman et al., 2018; Garcia et al., 2018).

### Gradually decreasing pulmonary blood volume during apnea

In our study, the PET-CT assessed pulmonary blood volume decreased 41% after 2 min of apnea and further to 67% after 4 min as assessed by CMRi. These results were supported by our echocardiographic study in which the pulmonary blood volume decreased by 26% after 2 min of apnea compared to rest, and further 36% after 4 min of apnea compared to 2 min of apnea. Hence, the BHD in our study had gradually decreased lung perfusion during maximum apneas similar to the Bottlenose dolphins and hereby a gradually altered V_A_/Q relationship as proposed by Garcia et al. (Garcia et al. 2018). We suggest the gradually altered V_A_/Q relationship, to serve as an oxygen conserving mechanism during dives, and the mechanism is suggested to be decreasing cardiac output as part of the human diving response but also reduced myocardial contractility due to progressive hypoxia (Kjeld et al., 2021c; Kjeld et al., 2009; Bing et al., 1975). Mijacika et al. demonstrated that pulmonary blood volume in the beginning of apnea decreased 530 ml and concluded that GPI causes lung vessel compression (Mijacika et al., 2017a; Mijacika et al., 2017a), and we do agree with this observation. However, the adaptation proposed by Garcia et al. in the Dolphin Bottlenose indicates that pulmonary blood volume also is decreasing to increase V_A_/Q and may compensate for decreasing oxygen stores during apnea. The results of our study indicate that this is the case in BHD, where pulmonary blood volume decreased by 26-41% after 2 min and by 48-67% after 4 min of apnea, respectively.

## Conclusion

In conclusion our findings differ from previous studies that showed similar lung gas transfer in elite BHD and matched controls. We conclude that 1) pulmonary blood volume may decrease during maximal apnea, which in turn increases V_A_/Q, to enhance alveolar oxygen delivery, and 2) the lower lung diffusion capacity observed in elite BHD compared to controls and increased lung perfusion during maximum apnea in our study are adaptations similar to diving mammals to tolerate decreasing tissue oxygenation.

## Perspectives

Our study demonstrated an increase in VA/Q during maximal apnea similar to air-breathing diving mammals (Garcia et al., 2018). Theoretically, these adaptations reduce gas exchange during diving and may protect against gas embolism and decompression sickness during diving. Hence, even SCUBA divers may benefit from training free diving to reduce risk of decompression sickness.

The results of the present study support the findings of our latest study: we suggest that during maximal apnea, blood is released from the lungs, spleen, and lower extremities and directed toward the hypoxia-sensitive abdominal organs as an oxygen conserving mechanism (Kjeld et al., 2024).

## Limitations

^15^O-H_2_O-PET/CT estimated pulmonary blood volume may differ from pulmonary blood volume estimated by CMRi and echocardiography. The results of the study by Harms et al., however, indicate that ^15^O-H_2_O-PET/CT and CMRi are highly correlated, but with systematic differences, (Harms et al., 2015), and the study by Margossian et al. indicates that left ventricle volumes determined by CMRi and echocardiography are comparable.

The membrane diffusion and the pulmonary capillary blood volume could not be retrieved from the body box spirometry, and these data may have confirmed our results from ^15^O-H_2_O-PET/CT, CMRi and echocardiography.

The number of subjects in this study is limited, and therefore future studies are needed to confirm our results.

## Data Availability

The data that support the findings of this study are all saved encrypted at hospital servers, but restrictions apply to the availability of these data, which were used under license for the current study, and therefore these data are not publicly available. Data are however available from the main author, T.K. upon reasonable request and with permission from Region Hovedstaden, Herlev Hospital Skejby Hospital and Rigshospitalet.
