# Supplementary material for: Pulmonary vascular adaptations to hypoxia in elite breath-hold divers
Source: Front Physiol. 2024 Jul 31;15:1296537. doi: 10.3389/fphys.2024.1296537 (PMC11318387; doi:10.3389/fphys.2024.1296537)
Supplement: Supplementary file 1 [file Table1.pdf]

Table S1 Subject characteristics.

	Breath-hold divers	Controls	P value
Number of subjects	9 males	8 males	NS
Age (years)	42 ± 3	36 ± 4	NS
Static personal best (seconds)	392 ± 21	N/A	NS
Dynamic pool personal best (meters)	163 ± 12	N/A	NS
Dynamic pool no fins personal best (meters)	133± 11	N/A	NS
Height (cm)	185 ± 3	183 ± 1	NS
Weight (kg)	79 ± 2	82 ± 2	NS
Body Surface area (Mosteller, m^2)	2.04 ± 0.03	2.04 ± 0.03	NS
Body Mass Index (kg/m^2)	23.6 ± 0.8	24.4 ± 0.6	NS
Maximal oxygen uptake (ml O2/min/kg)	47.5 ± 2.5	48.6 ± 2.1	NS
Hemoglobin (mmol/l)	8.9 ± 0.9	8.9 ± 0.3	NS
Ferritin ug/l	144.0 ± 19.1	141.6 ± 33.3	NS
Iron umol/l	16.9 ± 1.0	21.5 ± 1.8	NS

Basic morphometric data. Values are mean ± Standard error of the mean. NS: Not statistically significant.
