# Supplementary material for: Pulmonary vascular adaptations to hypoxia in elite breath-hold divers
Source: Front Physiol. 2024 Jul 31;15:1296537. doi: 10.3389/fphys.2024.1296537 (PMC11318387; doi:10.3389/fphys.2024.1296537)
Supplement: Supplementary file 2 [file Table2.pdf]

Table S2. Cardiac variables as evaluated with Echocardiographic imaging at rest in 6 elite breath-hold divers and 6 matched controls.

Values are mean ± Standard Deviations. EDV: end-diastolic volume; LV: left ventricular; ESV: end-systolic volume; EDV: end-diastolic. volume; RV: right ventricular; TAPSE: Tricuspid Annular Planar

Parameter	BHD	Controls	P-value
Left Atrium EDV, ml	20.2 ± 6.0	22.7 ± 5.8	NS
Mitral E/A-ratio	1.40 ± 0.36	1.51 ± 0.48	NS
LV Posterior Wall, mm	10.2 ± 1.5	10.7 ± 1.2	NS
LV Internal Diameter, mm	46.3 ± 2.9	50.0 ± 0.5	NS
Inter Ventricular Septum Diameter, mm	11.2 ± 0.10	11.5 ± 0.10	NS
LV EDV, ml	169.8 ± 6.4	161.7 ± 13.1	NS
LV ESV, ml	89.8 ± 5.1	95.5 ± 11.1	NS
LV Ejection Fraction, %	52.9 ± 1.7	59.2 ± 6.1	NS
LV Outflow Tract, mm	23.2 ± 9.8	23.7 ± 1.2	NS
Aortic Valve V_{max}, m/s	1.26 ± 0.23	1.31 ± 0.18	NS
Aortic Valve Velocity Time Integral, cm	29.4 ± 6.8	31.2 ± 4.0	NS
Aortic Valve Area, cm^2/m^2	3.98 ± 0.98	3.83 ± 1.01	NS
Aortic Ascendens, mm	28.7 ± 2.8	30.8 ± 4.9	NS
RV Internal Diameter, mm	32.6 ± 3.8	30.5 ± 0.5	NS
TAPSE, mm	27.3 ± 3.0	30.5 ± 0.5	NS
Tricuspid Valve max PG, mm Hg	25. 7 ± 2.5	26.4 ± 3.1	NS

Systolic Evolvement. PG: peak gradient. NS: not statistically significant.
